# Determinants of readiness to implement forensic patient-oriented research: a study of barriers and facilitators in a high-secure hospital

**DOI:** 10.3389/fpsyt.2024.1509946

**Published:** 2025-01-15

**Authors:** Cara Evans, Sevil Deljavan, Kayla Zimmermann, Kristy Allen, Elnaz Moghimi, Christopher Canning

**Affiliations:** ^1^ Waypoint Research Institute, Waypoint Centre for Mental Health Care, Penetanguishene, ON, Canada; ^2^ Department of Family and Community Medicine, Temerty Faculty of Medicine, University of Toronto, Toronto, ON, Canada; ^3^ Patient/Client and Family Council, Penetanguishene, ON, Canada; ^4^ Department of Psychiatry, Temerty Faculty of Medicine, University of Toronto, Toronto, ON, Canada

**Keywords:** patient-oriented research, participatory research, forensic mental health, high secure hospital, implementation

## Abstract

**Introduction:**

Forensic mental health care is intended to promote recovery and reintegration, but is often experienced by patients as punitive and aversive. Forensic patients are rarely engaged in research to explore what matters most to them, and little guidance exists on how this engagement may be facilitated. In this paper, we explore perceived determinants of readiness to implement forensic patient-oriented research in a high-secure setting.

**Methods:**

Following a period of engagement with staff and patients in the high-secure setting, we conducted interviews with 30 staff members (including clinicians, researchers, and hospital leaders) and five patients. We analyzed interviews using a thematic analysis approach. Coding was initially informed by the Consolidated Framework for Implementation Research, and subsequent iterations of analysis extended beyond this framework to explore patterns of meaning encompassing multiple implementation domains.

**Results:**

We identified three themes in our data: “Navigating a climate of distrust, discrimination, and restricted autonomy”; “Hearing and interpreting patient voices”; and “Experiencing a slow shift in the tide.” The first two themes represent potential challenges, including distrust and stigma; inherent restrictions in forensic care, and perceptions that patient autonomy threatens staff safety; patient fears of repercussions; and barriers to valuing and understanding patient voices. The third theme describes the ongoing shift towards patient-centredness in this setting, and participants’ interest in proceeding with forensic patient-oriented research.

**Discussion:**

Increased attention to relationship-building, trauma-informed principles, and epistemic injustice (i.e., unfair devaluing of knowledge) in high-secure settings can support the involvement of forensic patients in research.

## 1 Introduction

Forensic mental health care is intended to promote recovery and reintegration, but is often experienced by patients as punitive and aversive (1). Forensic patients are rarely engaged in research to explore what matters most to them, and little guidance exists on how this engagement may be facilitated. Given preliminary evidence for benefits of engaging forensic patients in research (2), we need to understand the barriers and facilitators to developing research partnerships with patients in high-secure forensic settings.

The forensic mental health system serves individuals at the intersection of the criminal justice and mental health systems (3). These include individuals who have been found Not Criminally Responsible or unfit to stand trial due to a mental illness at the time of their offense (4). The nature and quality of forensic care varies considerably worldwide (5). In more well-resourced systems, multidisciplinary teams—including psychiatrists, psychologists, nurses, social workers, and occupational therapists—work together to address the complex mental and physical health needs of forensic patients (6). Treatment and rehabilitation services in forensic settings are organized according to various levels of security (7). Higher levels of security include secure perimeters, locked units, and surveillance systems, with patients only allowed to leave under strict and supervised conditions. These services are designed to address patient needs while also ensuring patient, staff, and public safety (7). Patients’ length of stay is determined by ongoing assessments of risk and mental status, with attention to the balance of public safety and individual rights (8).

While intended to be recovery-oriented, forensic settings are often reported to be highly restrictive, coercive, and corrections-focused (3, 8–11). These environments tend to prioritize risk management at the expense of rehabilitation (1, 8). Patients commonly lack access to meaningful activities in their daily lives, frequently face stigmatization, and routinely encounter procedures and staff behaviours and attitudes that they perceive as demeaning or controlling (12–14). These conditions create substantial barriers to patient reintegration, contribute to poor patient outcomes, and perpetuate the marginalization of forensic patients (1, 12).

Meanwhile, patient-oriented research (POR) is grounded in valuing patients’ experiential knowledge (15, 16). POR draws on other participatory research approaches, such as participatory action research and community-based research, but is uniquely focused on improving health and healthcare systems (17). POR seeks to redistribute power and decision-making within research by engaging patients as equal partners throughout the research process, including when setting research priorities, conducting research, and translating findings into clinical practice and health policy (17–19). POR also includes active partnerships among researchers, healthcare providers, and health system decision-makers, transforming research into a shared endeavor (18, 20). The ultimate goal of POR is to utilize research findings to enhance patient outcomes (18, 21).

Although POR is becoming increasingly common in health services research, a scoping review (22) identified only two studies worldwide since 2016 that explicitly involved individuals with lived experience of forensic systems as members of the research team (23, 24). More recently, Dell and colleagues (25) conducted a POR project within a forensic psychiatric hospital, emphasizing the importance of relationship-building and managing power dynamics to ensure meaningful patient involvement in the research process. Additionally, a rapid review by Völlm et al. (2) summarized information from 23 articles published between 1980 and 2016 on engaging users of forensic mental health services as partners in research. The review highlighted several key areas relevant to the forensic context, including issues of power and vulnerability, practical difficulties, confidentiality and transparency, communication, and training (2).

The scarcity of studies that actively engage forensic patients as research partners can be attributed to various factors. One significant challenge lies in managing power dynamics within forensic settings (2). Principles integral to meaningful patient engagement, such as collaboration, power-sharing, and non-coercion, become more challenging when patients are detained or provided treatment against their will (2, 25, 26). Informed consent also presents particular complexities, as additional efforts are often required to ensure patients’ consent decisions are not influenced by external pressures (2, 27). Consent processes that are not tailored to meet patients’ literacy and cognitive needs can lead to disempowerment and perceptions of coercion (28, 29). These power dynamics can result in tokenistic involvement and hinder the development of trusting relationships (2).

Moreover, access to forensic patients willing to engage in research often necessitates negotiation with hospital staff who serve as gatekeepers (28, 30). There are frequent tensions between researchers and staff concerning security and risk management (30), further complicated by staff’s limited understanding of the value of research partnerships (2) and their inexperience in facilitating patient involvement in research (31). Even when access is gained, long-term patients may be accustomed to passivity and lack the motivation for empowerment or collaboration (25). Confidentiality issues are also relevant, as patients may be hesitant to disclose information to the institutions where they are detained or to their peers (2).

Despite these challenges, successfully engaging forensic patients in research can have enduring positive effects (2). It can foster a sense of empowerment, enhance self-esteem and feelings of self-worth, and build valuable skills (2, 32, 33). It also promotes strengths-based, recovery-oriented care (27) and supports the democratization of research practices and knowledge in secure forensic contexts (30, 34).

The present study is a component of a broader implementation project dedicated to establishing forensic POR (fPOR) practices at a psychiatric hospital in Canada with a dedicated secure forensic program. We aim to generate knowledge that can facilitate the tailoring of effective fPOR implementation strategies by exploring the determinants (i.e., barriers and facilitators) that may influence implementation. The research question is as follows: What are the perceived determinants to implementing fPOR in a secure forensic setting?

## 2 Materials and methods

### 2.1 Design

We draw on implementation science, the “study of methods to promote the systematic uptake of research findings and other evidence-based practices” (35), p. 1) to improve healthcare practices and patient outcomes. Implementation science can be used to understand factors that may influence the adoption of an evidence-based intervention in specific healthcare or public health settings, and to develop and test implementation strategies to enhance uptake (36).

In this context, the intervention of interest is fPOR, and the setting of interest is high-secure forensic care. Data collection and analysis were guided by the Consolidated Framework for Implementation Research (CFIR), a determinants framework that provides a taxonomy of barriers and facilitators to implementation (37). We chose the CFIR as a guiding framework due to its adaptability to diverse contexts and its ability to help in the identification of organizational and individual factors affecting readiness and implementation outcomes (38–40). According to the CFIR, five major domains (the intervention, inner and outer setting, the individuals involved, and the process by which implementation is accomplished) interact to influence implementation effectiveness (37). We operationalized the CFIR domains as follows:

1. Innovation: fPOR in secure forensic settings
2. Inner setting: the study site, a high-secure forensic program in an Ontario hospital
3. Outer setting: the federal and provincial mental health and criminal justice systems, health research community, and the general public
4. Individuals: the needs, capabilities, opportunities, and motivations of frontline staff, clinical and administrative leaders, researchers, and forensic patients
5. Implementation process: suggested strategies for implementing forensic POR

This operationalization of the CFIR informed the interview guide development. We used a framework approach to thematic analysis (41) to apply CFIR domains as initial deductive codes, develop additional inductive codes within CFIR domains, and finally to form themes that span these domains. Our analytic approach is described further below.

### 2.2 Setting

This study was conducted at a psychiatric hospital that provides specialized mental health, addiction and geriatric care, including a high-secure forensic program serving individuals found not criminally responsible or Unfit to Stand Trial. In this context, “high-secure” refers to long-term, locked units with no community access and no unsupervised visits from friends and family. Patients from the hospital’s high-secure forensic program are under the jurisdiction of a tribunal established under the Criminal Code of Canada. This tribunal holds hearings at least annually to review and determine patient dispositions, which can include: 1) detention orders for detainment in a facility; 2) conditional discharge order allowing community living under specified conditions; or 3) absolute discharge orders releasing the patient from oversight (4).

The high-secure forensic program provides structured group therapy and individualized care through comprehensive treatment plans developed and offered by a multidisciplinary team of practitioners. Security personnel work alongside care teams to facilitate a secure setting. The goal of the forensic program is to manage symptoms, reduce risk, and improve the quality of life for patients, ultimately supporting their rehabilitation and reintegration.

### 2.3 Sampling and recruitment

#### 2.3.1 Staff recruitment

We recruited participants from four groups of hospital staff: 1) hospital staff in leadership and decision-making roles within the high-secure forensic program or the hospital more broadly; 2) frontline clinical staff with current or recent (within the last two years) experience in caring for forensic patients at the hospital; 3) security personnel; and 4) research staff.

We sent all eligible staff a link to the electronic information and consent form via their professional email accounts. We then contacted consenting individuals to schedule interviews. To further promote the project and recruit staff for interviews, we presented this study at hospital leadership, research institute, and clinical meetings. Information about the study also appeared in the research institute newsletter and on the hospital’s internal website, and recruitment posters were displayed at the security office and on the high-secure units. Our study included 13 clinical and administrative leaders, 11 frontline clinical staff, one security team member, and five researchers.

#### 2.3.2 Patient recruitment

Patient recruitment efforts were led by a patient advocate, who is also a member of the research team (KZ). KZ is a member of a peer-led, non-profit organization that partners with organizations across the region to provide peer support and represent patient, client, and family voices in system decision-making. We used a purposive sampling approach to recruit current forensic patients enrolled in the hospital’s high-secure forensic program. This approach focused on patients who had existing relationships with patient advocates. Eligibility for participation was based on an expressed interest in the study, the capacity to participate (assessed based on patients’ wellness levels), and security clearance, as determined through consultations with patients’ clinical managers and patient advocates. We included five patients.

The recruitment process followed nearly a year of initiatives aimed at familiarizing patients with members of our research team and the concepts of research and POR. This prolonged engagement was essential for building trust and ensuring that patients felt comfortable and well-informed about the study. Consent procedures are described below under “Ethical considerations.”

#### 2.3.3 Information power

We used the concept of “information power,” or the sufficiency of qualitative data to address a research question, to assess our sample size (42). Information power is an alternative to saturation for study designs where theoretical saturation is not relevant (e.g., because theory is not being produced) and where an exhaustive description is not required to meet study aims (42). Malterud and colleagues argue that a smaller sample size is needed when the study aims are narrow, the sample is highly specific to the topic being addressed, the study is guided by existing theory, the data is rich, and the analysis focuses on a single case (42). This study focuses on fPOR in a specific programmatic area (forensics) within a specific institution (Waypoint); the interview sample comprises individuals deeply engaged with forensic care and research, who contributed rich interview data; and the analysis draws on a well-established implementation determinant framework. Our focused sample size of 35 participants aligns with similar, theory-guided implementation studies in healthcare contexts that use information power to inform sample size (43, 44).

### 2.4 Data collection

We collected data using semi-structured interview guides informed by the CFIR Interview Guide Tool (45) adapted to ensure relevance to forensic mental health settings and the diverse range of participants. The CFIR includes dozens of sub-constructs, and as such the CFIR Interview Guide Tool includes a lengthy list of questions. We only included constructs relevant to the current stage of implementation. We tested and iteratively revised the interview guide, and produced tailored interview guides for each participant category (Appendices A and B).

The research team’s principal investigator (CC), post-doctoral fellow (CE), and research analyst (SD) conducted staff interviews in-person or via videoconferencing according to participant preference. Each interview lasted 45 to 60 minutes and explored selected CFIR domains and their application to fPOR. For instance, interviews addressed anticipated benefits and challenges of fPOR, relationships among key stakeholders, values including patient-centeredness, and the potential impact of policies and regulations on the implementation of forensic POR. Thirty staff interviews were completed between November 2023 and January 2024.

KZ conducted patient interviews in-person at Waypoint in private rooms on patients’ respective units. Each interview lasted approximately 30 minutes. Interviews also used a semi-structured interview guide and addressed patients’ perceptions of the benefits and challenges of POR for themselves and other patients, how their personal experiences could inform research, and how researchers can demonstrate respect and value for patient perspectives. Five patient interviews were completed between May 2024 and August 2024. We conducted these interviews at a much later time than staff interviews, to allow sufficient time for building trust and establishing relationships with patients as described above.

All interviews were audio-recorded, and transcribed verbatim by a third-party service. Quotations included in this manuscript have been anonymized, and lightly edited (e.g., through removal of “filler words” such as “you know”) for clarity.

### 2.5 Data analysis

Due to our staggered approach to recruiting staff and patients as described above, we analyzed staff interviews earlier than patient data. CE and SD completed all coding, using NVivo Version 14.0.

To analyze staff interview data, we used a thematic analysis approach based on framework analysis (41). First, two coders familiarized themselves with the data through a review of transcripts. Second, we created an initial coding framework based on the CFIR. Two coders piloted the codebook on five transcripts through an iterative process that included independently applying the framework line by line, inductively adding codes, meeting to discuss discrepancies, and refining the approach. Third, a single coder then applied the updated framework to each of the remaining transcripts. Fourth, the coders charted data by analyzing data within designated CFIR domains and producing summary memos that were reviewed collaboratively. Finally, coders engaged in interpretation of the data. At this stage the two coders visually mapped their emerging analysis to identify four major thematic clusters, each of which spanned multiple CFIR domains. The entire research team subsequently reviewed and refined this analysis, and generated recommendations and implications.

Similarly, we analyzed patient interview analysis using a mixed inductive-deductive approach. Two coders applied a revised codebook based on the thematic analysis arising from staff interviews to three transcripts, and created additional inductive codes as needed before coding the remaining patient interviews. Any discrepancies along the way were discussed and resolved. We subsequently updated the thematic analysis to reflect findings from the patient interviews.

### 2.6 Ethical considerations

The Research Ethics Board at Waypoint approved this study. We created patient consent forms and interview guides in accessible, plain language, with input from PCFC to ensure that patients could fully understand the consent process and comfortably engage with the interview questions. Patient interviews were conducted by a PCFC patient advocate who is also a member of the research team and familiar with the patients. This familiarity enabled the advocate to assess the appropriateness of the timing for interviews, considering potential fluctuations in the patients’ capacity and wellbeing.

To protect confidentiality of all participants, data were anonymized and stored on a secure server on an encrypted institutional device, within a secure project folder accessible only to the research team. Potentially identifying details, such as references to specific units and healthcare providers, were removed from the data to prevent any recognition by peers within the organization. Staff participants received a $30 electronic gift card upon completing their interviews, and patient participants had $30 deposited into their hospital money accounts.

## 3 Findings

This section presents the key findings from our study, organized into three main themes. Each theme highlights challenges and opportunities associated with implementing and conducting fPOR in secure forensic settings. The themes offer insight into a pervasive distrust and discrimination that shapes interactions within the setting, the difficulties in centering and interpreting patient voices, the complexities around patient autonomy in restrictive environments, and a slow but steady shift towards more patient-centered approaches in forensic mental health settings.

### 3.1 Theme 1: navigating a climate of distrust, discrimination, and limited patient autonomy

Participants described the high secure forensic environment as one defined by isolation, suspicion, and discrimination. Patients and staff struggle with trusting one another, and feel vulnerable and exposed to various risks. Relationships between patients and staff are often strained, influenced by broader social stigmas and the historical context of the study site. This can result in the amplification of the inherently restrictive nature of forensic care.

#### 3.1.1 Distrust is pervasive

Distrust permeates the forensic setting, affecting both patients and staff. Patients often view staff with suspicion, feeling that the staff are “all on the same team,” which implicitly excludes them. This perception is reinforced by concerns that staff are not held accountable for their actions, as highlighted by a patient who noted that lawsuits target institutions rather than individual staff. Another patient echoed this, saying, “If they [staff] make a mistake, it’s not that big of a deal because it’s just coming out of [the government’s] pocket anyway.” This distrust extends beyond hospital staff to the broader mental health and criminal justice systems.

Staff also reported a sense of distrust on the units, and sometimes feel unprotected. Some staff believe that hospital leaders do not adequately consider the risks they face. One staff member recounted, “I’ve been in situations where there’s a very dangerous violent person that is very mentally ill and untreated. But by human rights we’ve got to get him out of his room for an hour and it’s like who is the person pushing for this?” Multiple staff described instances of physical harm or threat they encountered at work, sometimes in graphic detail, to underscore the point that those who do not work in the environment cannot fully appreciate its challenges. This belief extends to anyone not directly involved in frontline work, including researchers, with one staff member noting, “Unless you work on the floor, I think you are seen as upper management. And I think that you are looked down upon as you don’t get it.”

Distrust also manifests between different groups of staff, such as between allied health providers and nurses, or between newer and longer-tenured employees. In this environment, change is often perceived as a threat. One participant warned that “Most of the staff are in survival mode … so any changes, any new information, any added burden, all of that there’s going to be heavy, heavy resistance.” Another summarized this more bluntly: “We don’t like change, and we don’t like new people, and we don’t like anyone that isn’t being assaulted alongside with us.” In this regard, POR may be greeted with suspicion into what many described as an unpredictable environment.

There are deep historical and cultural roots to this distrust. Both patients and staff acknowledged the presence of broader social stigmas within the forensic setting related to mental illness and criminalization. As one staff member said, there is a persistent attitude that “most patients are here for a reason,” which leads to viewing patients through the lens of their past offenses. This dehumanization is compounded by the historical context of the facility, which stands on the site of a now-demolished institution known for unethical research practices in the 1960s and 1970s. The former facility existed up until the 2010s, and staff reported that practices were more correctional and punitive in nature. In acknowledgment of this history, one staff participant stated:

*I do think that the culture will play a significant contribution because we still have folks at [the hospital] that worked in the old [facility]…. And the reason I think it’s also applicable for staff that did not necessarily work at [the old facility] is they’re working directly with those … staff who are sharing their perspectives. We’re seeing changes in behaviour separate to what we give in orientation and training, because when they’re on the program being trained by particular staff, there are unconscious biases that are passed along that exist from [the old facility].*

These biases and distrust are critically important, because trusting relationships are foundational to POR. Participants emphasized the importance of an ongoing presence to build these relationships. Researchers were advised to commit to what one participant called “natural interactions in the natural environment.” For fPOR to be successful, these interactions will need to occur in a consistent and ongoing way to help build trust, as one staff participant said:

*I think trust is a huge factor when it comes to our forensic patients. [It] is something that takes … a really long time to build. And so being that constant presence, that familiar face, helps to build those relationships.*

Trust in research relationships, like in any other relationship, must be built through consistent interaction over time and founded on respect and honesty. As one patient described: “Just being treated equally is really the key, right.” Participants also highlighted that trust requires transparency. As described in Theme 2 below, patients have concerns about how information is shared and the potential consequences of participating in research projects. Building trust, then, requires clarity, as one staff participant described:

*I think that trust piece is huge. And so how are you going to potentially gather information? How are you going to keep that information? How are you going to use this? Are you going to use it against [patients]? Who’s going to have access to it?*

Participants also stressed that researchers should share findings of their study with patients and staff. They framed sharing back as an issue of “accountability” and “giv[ing] credit where credit is due,” and as a means of “work[ing] hand-in-hand together.” Trust could be built by ensuring that those participating in research are the first to learn about findings, and the first to benefit from these findings.

#### 3.1.2 Forensic care imposes limits on patient autonomy

In addition to trust, patient autonomy is a central concept of POR: patients need autonomy to choose to participate in POR and to make decisions about the conduct of research. Actualizing this autonomy will be challenging in forensic settings, which are inherently restrictive. Forensic services are legally mandated to detain patients until they are deemed safe and well enough for release, which often means that patients are involuntarily admitted and have little control over their circumstances. Many staff members acknowledged that the hospital operates as a “policy heavy organization” that prioritizes safety and security above all else due to the high-risk nature of patients with acute mental health needs and histories of violent behaviour. One staff participant said that patients at the hospital are ones “that no other hospitals either want or can handle.” Another staff participant highlighted the uniqueness of the hospital’s challenges:

*We have to be well equipped and … able to manage clients and their specific needs, which can be very difficult at times. So, it’s unique in the sense that we have to deal with a lot of stuff that other hospitals don’t. And there’s situations … Not that situations don’t get very violent at other hospitals, but we deal with it at extreme levels. I’ve seen patients literally break out of seclusion rooms, bashing TVs through the glass, and using them, a piece of metal, as weapons. That doesn’t happen every day at a medium secure hospital.*

In response to these risks, patient freedom is heavily restricted and governed by a web of laws and regulations, detailing, as one staff participant said, “what they need to do, when they need to do it, and why they need to do [it].”

However, some participants disagreed with the restrictive practices. Some patients perceive these restrictions as excessive, questioning whether restrictions are truly necessary for safety or if they could be relaxed to allow more autonomy. As one patient said, “Well, I think we all should look into what we do every day in these places … what we should be allowed to do, and what we’re restricted from for no reason.” Some staff participant also expressed concerns about restrictive practices, with one stating, “There are many regulatory and legal obligations that actually contradict what we would think would be best interest for our clientele.” Another recounted a situation where a patient questioned the fairness of collective consequences for individual actions:

*I was on a unit for one of our meetings, and a patient kept asking, for better or for worse, ‘Why am I being punished for other people’s behaviors? Why am I being punished for other people’s behaviors? So, someone else did this, but you’re taking this away from me. So, you’re taking this away from me because someone else did this.’ And, in truth, that is a very fair question.*

One participant, who held a research role, described forensic care as “totalizing” and capable of stripping “anyone’s freedom away at any moment.” The concerns around restrictiveness suggest that the boundaries are blurred between inherently restrictive qualities of forensic care, and usual (but modifiable) practice.

A key driver of additional restrictions to autonomy derives from the distrust described above. There is a prevailing belief among some staff that patient-centeredness conflicts with staff safety. A few participants described a “pendulum” that had swung too far in favour of patient rights, leaving staff feeling unsupported and at risk. One staff member remarked,

*I think [the hospital] is patient-centered, to a fault. I think that [old facility] was not patient centered. I think that it was an abusive environment. I’m sure we’ve all heard those stories. And I think that legally, we swung so far to patient right[s], patient-centered care … Staff feel that they have no opinion on their own safety … Our decisions are often based around, all right, he hasn’t come out of his room in seven days. He’s got to come out. Who’s comfortable? Oh, none of us are really comfortable, but he’s got to come out of his room. I think everything we do is patient centered.*

Without reconciling safety and autonomy, it will be difficult to gain staff buy-in for POR. Staff are unlikely to support a practice that they could perceive as a direct threat. However, some participants recognized that a trauma-informed approach could potentially reconcile patient-centeredness with safety. For instance, one staff participant noted a trauma-informed approach “would diminish the threats and the ongoing behaviours that we’re so afraid of in the first place.” This points towards avenues for aligning POR with the safety needs of both patients and staff.

### 3.2 Theme 2: hearing and interpreting patient voices

POR aims to centre and elevate patient voices, but this approach is more challenging in a high secure forensic context. Patients may be reluctant to speak out due to fear of repercussions, and when they do speak, their voices may be devalued or misunderstood by others.

#### 3.2.1 Patients fear repercussions

Patients expressed concerns about the potential consequences of voicing their opinions, particularly if those opinions are critical of the institution. This poses a clear and obvious challenge to participating in research and quality improvement, as gaps or problems are a typical starting point for research questions. Patients’ fear stems from the knowledge that anything they say could be documented and reported to the tribunal that determines their progress and privileges. One patient described the constant vigilance required to avoid negative consequences:

*They chart everything you do. Did you eat today, did you sleep to today, did you shit today? How are you feeling? And they use that against you, and then they re-word it, and it goes to the higher-ups or [the review board] or wherever you’re going to. And then it becomes some big problem … Maybe you should be locked in your room and punished and lose your levels … So, you’re always on guard and on fear.*

To “lose [one’s] levels” means losing privileges such as access to spaces within the hospital. The tribunal also determines when patients are eligible to move from the highly restrictive secure setting in which this study took place, to lower security units and eventually the community. Given these stakes, patients may choose to remain silent rather than risk their freedoms. As one staff participant stated,

*[Patients] don’t have a lot of trust in nurses or in the system because everything they say does get documented. And they have a history of seeing their own sort of expressions being heard at the [tribunal] … You know, if they start running down the hospital’s practices, or whatever the case may be, they’ll probably be guarded and say, “I can’t say anything bad about the hospital because I know that it’ll come back to me. It has before.”*

Centering forensic patient voices in POR might be pitted against patients’ interest in preserving their already limited freedoms. Some participants acknowledged that research activities would not be reported to the tribunal outside of safety-related concerns. Nevertheless, this distrust and fear of repercussions will likely be a significant barrier to patient participation in research.

#### 3.2.2 Patients’ voices must be valued and understood

It is not enough for patients to speak. POR demands that patient voices are heard, understood, and taken seriously. However, both patients and staff suggested that patient perspectives are often dismissed. Moreover, researchers may lack skills to effectively interpret patient voices, as one patient participant remarked: “I think that would probably be the hardest thing: for a patient to explain themselves in a way that a non-patient might understand.”

Patient participants reported feeling dismissed by staff when they do speak up, including a discounting of patient viewpoints due to mental health-related discrimination. One patient lamented, “Nobody’s going to believe some mental patient. Oh well, you’re just insane.” As another patient said: “[Patients are] not listened to. They’re considered, oh, we’re above you, we have more seniority … So it doesn’t matter if we’re wrong, we’re still right because we’re the staff, we’re in charge, and we have the right to do wrong things to you.”

This dismissal extends to staff who may also be skeptical of the value of patient voices, often citing concerns about manipulation and perpetuating an “us vs. them” ethos described in Theme 1. Indeed, some staff participants expressed skepticism towards the value of patient voices in some contexts. This skepticism was often framed in terms of worries about “manipulation”:

*I think the big one is there’s a manipulation. Like I see this all the time with the patient satisfaction survey. Which I also believe in. We need these things. But the only people that fill them out or that want to participate are the ones that hate everything, right? We have a very hard time getting the people who are kind of okay or might have some good suggestions.*

Another staff stated that research “would become like a bit of a complaint fest … It’d be a lot of manipulation involved.” In this regard, several staff participants advised clinical screening for appropriateness of involvement in research:

*You would also have to do like I think tests or work with a psychometrist to find the right patients for this. That have like the right intentions and have the kind of the IQ to follow along, and have like the capabilities to get through it. Yeah, there would probably have to be some sort of evaluation in order to find the right person for this.*

However, interpreting all patient complaints as a form of manipulation or a clinically-relevant problem inhibits patients’ ability to communicate concerns—including what one staff participant described as “totally reasonable complaints about the system.”

In addition to the willingness to listen, there is a need for researchers to possess the skills required to interpret the complex communication styles of forensic patients, particularly those with intellectual disabilities or personality disorders. One participant highlighted that de-escalation skills are needed to understand patient voices when emotional intensity is high: “I imagine some people that participate are going to be upset with the system. And being able to de-escalate some of those complaints and be[ing] able to get past that to some productive kind of feedback, I think will be very important.”

Effective communication is relational, requiring ongoing interaction and context to understand the messages being conveyed. One participant described how an ongoing relationship can facilitate communication with patients with intellectual disabilities by providing context to understand the communicative function of behaviour:

*I’m not saying they [patients with intellectual disabilities] should not be involved in research, but I’m implying the manner in which you obtain data from the patient should not be a didactic kind of question answer because they don’t do well with question/answers. A bit more longitudinal observation can actually provide a lot more of what the patient really wants. To hear their voice, it’s more of a behavioural observation than kind of a question/answer.*

Meanwhile a patient participant further described how humility can create a relational context for communication:

*If I don’t know nothing about nothing, and you coming in as a researcher don’t know anything, or we’re looking for the common ground, well, it’s a learning experience for both of us now. You grow together…. Because if one comes in with the attitude that, well, I know this stuff, I’ve been studying this, again, the other guy just wants to hide in the corner, right?*

There are layers of complexity involved in centering forensic patients’ voices in fPOR, including patients’ perceptions of the risks of speaking, and researchers’ openness to—and skillfulness in—understanding what patients have to say. Effective communication will be necessary to support other foundational elements of POR, including active collaboration and patient leadership.

### 3.3 Theme 3 : Experiencing a slow shift in the tide

While some participants described the shift towards patient-centeredness as gradual and incomplete, there is a growing enthusiasm for POR. This shift represents an important opportunity to address the challenges of POR in a forensic context.

#### 3.3.1 Attitudes towards patient-centeredness are changing

Participants noticed a shift at the hospital towards patient-centered care, although challenges remain. The hospital aspires to patient-centered, recovery-oriented care, with staff providing examples such as changes in “room extractions” and involving patients in policy development. One participant remarked, “It would be inconceivable to have this meeting, talking about [POR], maybe 15 years ago.”

A clinical leader also observed a cultural shift from a security-first mentality to a more balanced approach:

*…historically speaking, especially those of us who are up at [old facility], the mentality and the way that we were taught was that you’re security. Yes, you are a nurse, but you are security, and that comes first and foremost … the patients didn’t really have too much of a say in what happened day-to-day. We would tell them when to wake up, we would tell them when to eat, we would tell them when they were going outside. And not that that has really shifted because there is still some structure that’s needed. But I do think now you can come to us and say, Well, I don’t like to wake up in the morning. And so we’ll work with the patients to try and figure out like best case scenario, like, how do we get the things that we need to get done and things that you want to do? How do we make that happen without disrupting what you need from us?*

Despite this progress, fully realizing patient-centeredness is hindered by the limitations of high-secure forensic settings and staff burnout. One participant noted,

*I think on a daily basis, in a staff that’s burnt out, and a staff that, you know, might take verbal or physical abuse on a regular basis as a manifestation of somebody’s mental illness … I think it’s very, very, very difficult to see and be part of that every day, and see past that, and be completely patient centered.*

However, there is optimism that the hospital is “getting there” in terms of patient-centeredness.

#### 3.3.2 Many believe fPOR will have a positive impact

Participants generally believed fPOR aligns with broader shifts towards patient-centered and recovery-oriented care in forensic settings. One noted, “[fPOR] matches the philosophy of recovery care … It brings a sense of hope and purpose.” POR provides patients with a voice and autonomy, particularly in a setting where choices are often limited. As one patient stated, “I’m voicing my feelings right now. And you don’t get a lot of people you can do that with in an establishment like this.”

Staff highlighted the transformative potential of patient involvement in research, noting it could foster confidence, self-esteem, and a sense of purpose. As one staff participant said:

*I think if I put myself in the patient’s perspective, if you’re studying something that’s interesting to me because I care about it, likely it’s something that I also think will help me or help my family. And so, unlocking what they feel might be exactly what they need to heal and grow, and become an active and contributing member of society. And also might help them, especially in our area, just feel more engaged and having more purpose in their space.*

Participants noted that everyone in high-secure forensic settings would need some understanding of the nature and purpose of fPOR. As one staff participant noted, “the first milestone is just getting people to understand what POR is.” Once that understanding is in place, participants believed that fPOR could generate excitement. For some staff participants, advocating for groups that are marginalized or “overlooked” is a key motivator for involvement in fPOR. One researcher noted: “I want everyone to have an equal space at the table, and equal opportunity to share their voice. So that’s an intrinsic motivator for me personally.” Another staff participant stated that, “So I think the motivation would be that we could do something really groundbreaking, and we could be kind of helpful to, you know, really look at what are we doing, and why are we doing it, and is it working?”

Participants acknowledged that patients might prioritize different aspects of care compared to staff and researchers. One patient highlighted the diversity of perspectives across the hospital, stating that everyone’s “point of view,” “knowledge,” and “stance” differs. Another patient echoed this sentiment, saying: “I think it’s cool for the patients to be involved because there could be different topics or different objectives that could be dealt with.” One staff member agreed:

*I think that our patients and those that are within our care are likely best positioned to help guide us in areas of interest, areas that are gaps in the care that they’re receiving. I think what may be of interest to a researcher may not necessarily be what’s of interest to a patient.*

By aligning research with what is “actually meaningful to the patients,” many participants believed that fPOR can lead to more relevant research and could ultimately improve patient outcomes.

## 4 Discussion

In this study, we identified perceived barriers and facilitators to implementing POR in a high-secure forensic setting. These include pervasive distrust between patients and staff, and among staff groups. Patients also described experiencing discriminatory beliefs and behaviours from staff. Secure settings and the inherently restrictive nature of the context present formidable challenges to enabling patient autonomy. However, a further challenge was found in staff beliefs that patient centeredness necessarily comes at the expense of staff safety. While POR aims to centre patient voices, both staff and patients noted barriers to patients speaking, being heard, and being understood. Patients may choose not to speak out of concerns that their contributions may be relayed to the tribunal that determines their disposition orders. Meanwhile, staff may downplay the meaning of patient expression—particularly when critical—and researchers may lack skills to navigate patients’ communication needs and styles. However, despite these barriers, participants pointed out positive changes in the culture of forensic services over time and expressed optimism about POR. In light of these findings, we suggest that implementing POR in forensic settings will require a number of contextually-sensitive approaches, including: building relationships with staff gatekeepers; adopting trauma-informed principles; practicing transparency around reporting; attending to epistemic justice; and leveraging growing interest and enthusiasm.

Forensic staff are key gatekeepers, and they may distrust researchers. Deliberate, transparent, and longitudinal relationship building between POR teams and clinical staff will be necessary to build trust over time. Moreover, frontline staff in our study reported feeling threatened by patient centredness and patient autonomy. This perceived threat will itself be a critical barrier to staff engagement. The literature on trauma-informed care may offer one way through this perceived deadlock, as two participants noted. Trauma-informed principles are founded in a recognition of the high prevalence and pervasive impacts of psychological trauma, and include transparent communication, collaborative relationships, and prioritizing the safety of both service users and providers along with attention to the broader, structural context in which psychological trauma occurs (46). Trauma-informed care has been found empirically to reduce restraint and seclusion in inpatient mental health settings (47). Working with frontline staff to adopt a trauma-informed research approach may help to reframe patient autonomy as a safety-promoting factor rather than a threat; it may also support less stigmatizing understandings of patient behaviour. It is also important to note that when trauma-practices are misunderstood or co-opted, practices labelled as trauma-informed may merely replicate prior ways of working, or even enact new harms (46, 48, 49). POR practitioners will need to carefully consider these risks, particularly given structural barriers to fulsomely enacting principles like collaboration in high-secure settings.

Addressing barriers to hearing and interpreting patient voices will further require a multi-pronged approach. Patient fears of expression can be managed through clear and consistent communication around reporting, and what will and will not be documented. POR researchers can also draw on the literature on epistemic injustice when seeking to amplify forensic patients’ voices. Epistemic injustice is a concept that was initially articulated by Miranda Fricker to describe ways that knowledge is unfairly discredited or discounted. “Testimonial injustice” occurs when a person is treated as incapable of knowing due to prejudices against them; “hermeneutic injustice” occurs when a person’s interlocutor (or the dominant culture) lacks an interpretive framework to make sense of particular experiences or ways of knowing (50). In our study, staff dismissal of patient testimony as mere complaint or manipulation constitutes testimonial injustice—a form of injustice that is particularly well-documented in the realm of mental health, given stigmatizing beliefs about the credibility of people with mental illness (51). Meanwhile researchers’ lack of skills to understand patients’ communication may be a form of hermeneutic injustice. Participatory research has been argued to hold potential as a corrective to epistemic injustice (52, 53), suggesting that POR may contribute to challenging this inequity. However, other scholars have noted that participatory research can also replicate existing hierarchies of knowledge (54, 55), as the premise of participatory approaches requires a distinction between academic researchers and an “other” (55). Groot and colleagues (54) argue that meaningfully addressing epistemic injustice within participatory research requires thoughtful attention to relational dynamics, and suggest that participatory research teams must respond to uncertainty, change, and threat by enacting care and connection. We propose that strong relationships within the POR team may support initial implementation in an unjust context, while sharing the process and products of POR research can reduce epistemic injustice in the forensic setting over time.

While secure forensic settings represent a relatively new terrain for POR, core objectives and principles will still apply. The challenge will come in operationalizing these objectives and principles within the substantial constraints that this context imposes. The “shift in the tide” of attitudes that we found aligns with much of the POR literature broadly: many scholars have argued that partnering with patients has the potential to transform health research by increasing its relevance and impact (15, 19, 56). Participants’ optimism in the face of challenges suggests that the time is ripe to begin this work—and that while trust and collaboration may pose a challenge, allies and champions will be found along the way.

### 4.1 Strengths and limitations

A strength of this study is the breadth of perspectives represented. Participants included patients, nurses, allied healthcare providers, clinical and security leaders, researchers, and hospital directors and executives. Moreover, this study was conducted following intentional relationship building with both patients and staff. Research team members attended meetings and events and spent unstructured time on the forensic units, seeking to build familiarity with and trust in the research team. We believe that this relationship building resulted in richer data, as evidenced by participants’ detailed and candid responses. Finally, using CFIR to guide data collection and initial analysis helped point towards factors known to affect implementation processes—while subsequent analysis across CFIR domains deepened our analysis and allowed for exploration of complexity.

Our study also has important limitations. Patient participants are not representative of the full diversity of forensic patients in the study site. In particular, we did not interview patients with intellectual disabilities, who comprise a significant proportion of this population. As well, despite efforts to build relationships, it is important to note the general distrust of research within the institution. Participants are likely to be those who are more comfortable with the research process, and as such do not reflect the full range of viewpoints.

## 5 Conclusion

There is momentum building towards fPOR in secure forensic settings. Increased attention to patient-centeredness, shared decision-making, relational security, and trauma-informed care in this context lays a critical foundation that can support the involvement of forensic patients in research. With that said, forensic POR presents formidable barriers.

As described above, overcoming distrust, fostering understanding, and enabling autonomy will hinge on structural competence and solid relationships. fPOR researchers need an astute understanding of the structures that organize forensic patients’ lives—including formal policies as well as broad forms of oppression and marginalization. It will be critical for researchers to distinguish between the barriers that are relatively immovable, such as the mandated nature of treatment, and aspects of culture and attitude that are subject to change. Where change is possible, it will be possible through relationships. These relationships with patients, clinical staff, security staff, leaders, and other stakeholders must be built over time and continually re-affirmed through careful and caring navigation of challenges.

As research teams take up fPOR approaches in secure forensic settings, the field would benefit from well-documented studies of this process. The present paper explored perceived determinants; future research can describe approaches to address barriers, including detailed descriptions of strategies and their intended and unintended effects. Future research should include patient perspectives on involvement in research, and should explore outcomes for patients, family members, and other members of research teams, the secure settings in which fPOR takes place, and the broader field of forensic mental health.

## Data Availability

Data will not be made publicly available, in order to protect confidentiality of participants. Requests to access the datasets should be directed to ccanning@waypointcentre.ca.
